# Safety assessment of Osilodrostat: The adverse event analysis based on FAERS database by means of disproportionality analysis

**DOI:** 10.1371/journal.pone.0329088

**Published:** 2025-08-07

**Authors:** Lijun Li, Wanchen Zhao

**Affiliations:** 1 Department of Pharmacy, the Second Affiliated Hospital, University of South China, Hengyang, China; 2 Hengyang Medical School, University of South China, Hengyang, Hunan, China; 3 Hunan Provincial Key Clinical Laboratory of Basic and Clinical Pharmacologlcal Research of Gastnointestinal cancer, the Second Affiliated Hospital, University of South China, Hengyang, Hunan, China; 4 Department of Nursing, Peking Union Medical College Hospital, Chinese Academy of Medical Sciences, Beijing, China; Jordan University of Science and Technology Faculty of Medicine, JORDAN

## Abstract

**Background:**

Osilodrostat is a medication recently approved for the treatment of Cushing’s syndrome. However, there is a current dearth of large-scale studies on the adverse events associated with Osilodrostat. Consequently, this study aims to comprehensively evaluate these adverse events using data from the FDA Adverse Event Reporting System (FAERS).

**Methods:**

A disproportionality analysis was utilized to identify signals of adverse events linked to Osilodrostat. Furthermore, a Weibull distribution analysis was conducted to evaluate the temporal evolution of adverse events, and subgroup analyses were performed. The Wilcoxon test was applied to investigate differences in the temporal patterns of adverse events across different genders.

**Results:**

A total of 1,078 cases related to Osilodrostat were identified, including 3,744 adverse events. The most frequent and severe signals of adverse events were investigations, off-label use, fatigue, nausea, and adrenal insufficiency. The median time to onset of adverse events related to Osilodrostat was 52 days after starting the medication. There was a gender difference in the median time to onset of adverse events, with a median of 15 days for males and 34 days for females.

**Conclusion:**

This study provides a comprehensive evaluation of adverse events related to Osilodrostat, confirming some known side effects and revealing other potential risks. This information offers valuable insights for the clinical application of Osilodrostat.

## Introduction

Osilodrostat functions as a dual inhibitor within the biosynthesis pathways of glucocorticoids and mineralocorticoids. It achieves this by inhibiting the production of CYP11B1, which is the catalyst for the final step in cortisol synthesis. Additionally, it can also impede the production of CYP11B2, the enzyme that catalyzes the conversion of corticosterone to aldosterone [1]. In early 2020, Osilodrostat received approval from both the European Medicines Agency (EMA) and the U.S. Food and Drug Administration (FDA) for the treatment of Cushing’s syndrome (CS) [2,3]. Given its rapid onset of action and long half – life, it is emerging as a new ray of hope in the treatment of Cushing’s syndrome for patients who are ineligible for pituitary surgery or for whom such surgery has proven ineffective [4].

With the increasing acceptance and expanding application of Osilodrostat, it is crucial to explore the drug-related adverse events (AEs) associated with it. Although several studies have investigated these AEs, but all of them are faced with challenges such as small sample sizes and inconsistent findings. For instance, the studies by Pivonello [5] and Fleseriu [6] have identified nausea, headache, fatigue and adrenal insufficiency as the most common AEs related to Osilodrostat. In contrast, other studies have indicated that diarrhea, hypokalaemia, muscle spasms, vomiting [7], arthralgia [8], decreased appetite [9], gamma-glutamyl transferase increase, nasopharyngitis [10], depression [11] are the most prevalent AEs. Currently, the safety information regarding Osilodrostat mainly stems from case reports, clinical trials, and meta-analyses. However, these studies often target on the specific populations or include relatively limited sample sizes and selection criteria. As a consequence, there is a dearth of comprehensive safety data from large samples and real-world cohorts [12].

The FDA Adverse Event Reporting System (FAERS) holds the potential to overcome the existing limitations in research on drug-related AEs associated with Osilodrostat. Numerous prior studies have utilized the FAERS database to explore drug-related AEs [13–17]. FAERS, a publicly accessible, large-scale database managed by the FDA, is specifically designed to facilitate the post-marketing safety surveillance program for drugs and therapeutic biologics. Since 2004, it has encompassed all AE reports and medication error reports submitted to the FDA by healthcare professionals, consumers, and manufacturers [18].

Therefore, the present study is aimed at evaluating the safety of Osilodrostat by analyzing data from the FAERS database. The intention is to offer a reference for the clinical application of Osilodrostat, facilitating more informed decision – making in its use within the medical field.

## Materials and methods

### Data sources, management, ethics statement and study design

**Fig 1:**
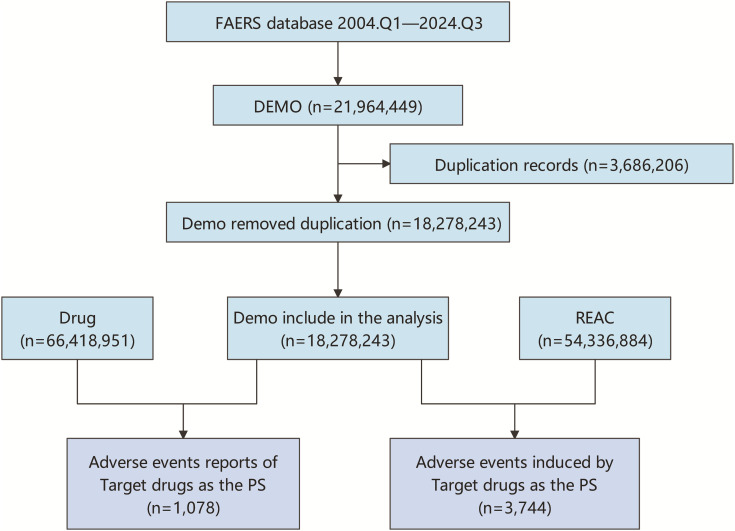
Flowchart for Osilodrostat FAERS adverse events.

The raw data for this study was obtained from the FAERS database. All adverse event (AE) reports in which Osilodrostat was identified as the primary suspect drug were retrieved from the database, spanning from the first quarter of 2004 to the third quarter of 2024 in the database. Deduplication was carried out in accordance with the method recommended by the FDA [19]. To eliminate duplicate reports, the PRIMARYID, CASEID, and FDA_DT fields were selected from the DEMO table. The reports were sorted by CASEID, FDA_DT, and PRIMARYID. For reports with the same CASEID, the one having the largest FDA_DT value was retained. In cases where both CASEID and FDA_DT were identical, the report with the largest PRIMARYID value was kept. Subsequently, the AEs’ names in the FAERS database were encoded using MedDRA 27.1. Ethical approval was not required for the study involving humans in accordance with the local legislation and institutional requirements. Written informed consent to participate in this study was not required from the participants or the participants’ legal guardians/next of kin in accordance with the national legislation and the institutional requirements. The detailed flowchart of the study design is shown in Fig 1.

### Statistical analysis

Descriptive analysis was utilized to delineate the fundamental demographic data of the patients and the distribution of target drug’s AEs across different system organs. Four disproportionality analysis techniques were implemented for detecting signals of AEs related to Osilodrostat: the reporting odds ratio (ROR) [20], the proportional reporting ratio (PRR) [21],the multi - item gamma Poisson shrinker (MGPS) [22], and the Bayesian confidence propagation neural network (BCPNN) [23]. When two or more analysis methods were combined for detection, AEs that met the positive threshold of all the analysis methods used were considered potential AE signals. The interval between the occurrence of the AEs recorded in the DEMO file and the initiation of Osilodrostat treatment recorded in the THER file was defined as the onset time for Osilodrostat-related AEs. The Weibull distribution was applied to model the changes in the incidence of AEs over time. All statistical analyses were conducted using SAS 9.4.

## Result

### Descriptive analysis

**Fig 2:**
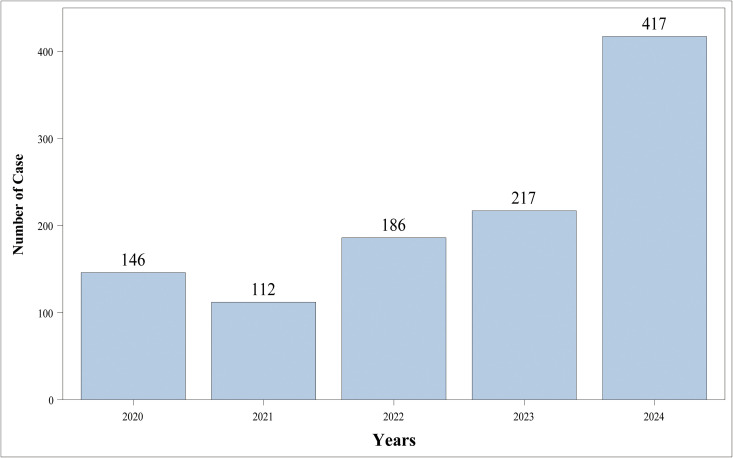
The distribution of the data in the FAERS database.

A search was conducted for data in the FAERS database from the first quarter of 2004 to the third quarter of 2024 (Fig 2). After data cleaning, a total of 1078 Individual Case Safety Reports (ICSRs) related to Osilodrostat were retrieved, containing 3744 AEs. The clinical characteristics of these reports were shown in Table 1. Among all the included patients, females constituted 12.62% and males accounted for 4.55%. The mean age of the included patients was 49.66 ± 16.23 years, with the majority of patients in the age group of 45–64 years, accounting for 6.12%. The majority of reporters were consumers, accounting for 78.29%. Regarding geographical distribution, the United States had the highest number of occurrences and reports of Osilodrostat-related AEs (occurrences:86.09%, reports:85.44%)(S1A Fig). Similarly, North America was the continent with the most occurrences and reports of AEs related to Osilodrostat (occurrences:86.09%, reports:85.44%). In terms of the severity, 59.83% of the reports were classified as serious, while the remaining 40.17% were non-serious. As for the outcomes of the AEs, the occurrence of other serious medical events was the most prevalent outcome, accounting for 32.19%, followed by hospitalization (29.59%), death (9.93%), life-threatening (1.39%), and disability (0.28%)(S1B Fig).

**Table 1:** The clinical characteristics of all the included reports.

Clinical characteristics	Number of cases (%)
Gender	
Female	136 (12.62)
Male	49 (4.55)
Not specified	893 (82.84)
Age	
<18	2 (0.19)
18-44	58 (5.38)
45-64	66 (6.12)
≥65	31 (2.88)
Not specified	921 (85.44)
Age (quantitative)	
N (Missing)	157 (921)
Mean (SD)	49.66 (16.23)
Median (Q1, Q3)	49.00 (37.00, 62.00)
Min, Max	16.00, 87.00
Year of report	
2020	146 (13.54)
2021	112 (10.39)
2022	186 (17.25)
2023	217 (20.13)
2024	417 (38.68)
Reporter	
Consumer	844 (78.29)
Pharmacist	78 (7.24)
Physician	156 (14.47)
The continent in which the AEs occurred	
North America	928 (86.09)
Europe	73 (6.77)
Asia	51 (4.73)
South America	17 (1.58)
Oceania	9 (0.83)
The country in which the AEs occurred	
United States of America	928 (86.09)
Japan	47 (4.36)
France	38 (3.53)
Colombia	16 (1.48)
Poland	12 (1.11)
The continent of the reporting country	
North America	921 (85.44)
Europe	78 (7.24)
Asia	51 (4.73)
South America	17 (1.58)
Oceania	9 (0.83)
Not specified	2 (0.19)
The country from which the reports come	
United States of America	921 (85.44)
Japan	47 (4.36)
France	41 (3.80)
Colombia	16 (1.48)
Poland	12 (1.11)
The severity of the reports	
Serious	645 (59.83)
Non-serious	433 (40.17)
Outcome	
Life-threatening	15 (1.39)
Hospitalization – initial or prolonged	319 (29.59)
Disability	3 (0.28)
Death	107 (9.93)
Other	347(32.19)

### Distribution of the AEs at the system organ class (SOC) level

A total of 26 SOCs were associated with the AEs related to Osilodrostat. The top 5 SOCs with the highest report frequencies were General disorders and administration site conditions (n = 625, ROR 0.95, PRR 0.96, IC −0.06, EBGM 0.96), Investigations (n = 441, ROR 2.04, PRR 1.92, IC 0.94, EBGM 1.92), Gastrointestinal disorders (n = 440, ROR 1.43, PRR 1.38, IC 0.47, EBGM 1.38), Injury, poisoning and procedural complications (n = 370, ROR 0.95, PRR 0.96, IC −0.06, EBGM 0.96), and Nervous system disorders (n = 313, ROR 0.98, PRR 0.98, IC −0.03, EBGM 0.98). In addition, the SOCs that were significantly associated with the AEs of Osilodrostat and met the criteria of ROR, PRR, BCPNN and MGPS, four detection methods, were investigations (n = 441, ROR 2.04, PRR 1.92, IC 0.94, EBGM 1.92), Gastrointestinal disorders (n = 440, ROR 1.43, PRR 1.38, IC 0.47, EBGM 1.38), Musculoskeletal and connective tissue disorders (n = 224, ROR 1.17, PRR 1.16, IC 0.21, EBGM 1.16), Metabolism and nutrition disorders (n = 160, ROR 2.01, PRR 1.97, IC 0.98, EBGM 1.97), Endocrine disorders (n = 118, ROR 12.80, PRR 12.43, IC 3.63, EBGM 12.42)and Surgical and medical procedures (n = 72, ROR 1.44, PRR 1.43, IC 0.52, EBGM 1.43) (Table 2).

**Table 2:** The distribution of the adverse events at the system organ class (SOC) level.

SOC	Number of cases	Proportion (%)	ROR(95% CI)	PRR(95% CI)	IC(IC025)	IC(IC025) (Noren)	EBGM(EBGM05)
General disorders and administration site conditions	625	16.69	0.95(0.87,1.03)	0.96(0.89,1.03)	−0.06(−0.19)	−0.06(−0.20)	0.96(0.88)
Investigations	441	11.78	2.04(1.85,2.25)	1.92(1.75,2.09)	0.94(0.79)	0.94(0.78)	1.92(1.73)
Gastrointestinal disorders	440	11.75	1.43(1.30,1.58)	1.38(1.27,1.51)	0.47(0.32)	0.47(0.31)	1.38(1.25)
Injury, poisoning and procedural complications	370	9.88	0.95(0.85,1.06)	0.96(0.87,1.05)	−0.06(−0.22)	−0.06(−0.24)	0.96(0.86)
Nervous system disorders	313	8.36	0.98(0.87,1.10)	0.98(0.88,1.09)	−0.03(−0.19)	−0.03(−0.21)	0.98(0.88)
Musculoskeletal and connective tissue disorders	224	5.98	1.17(1.02,1.33)	1.16(1.02,1.31)	0.21(0.01)	0.21(−0.01)	1.16(1.01)
Infections and infestations	206	5.50	1.05(0.92,1.21)	1.05(0.92,1.20)	0.07(−0.13)	0.07(−0.16)	1.05(0.91)
Metabolism and nutrition disorders	160	4.27	2.01(1.72,2.36)	1.97(1.69,2.29)	0.98(0.74)	0.97(0.71)	1.97(1.68)
Psychiatric disorders	157	4.19	0.73(0.62,0.86)	0.74(0.64,0.86)	−0.43(−0.66)	−0.43(−0.69)	0.74(0.63)
Skin and subcutaneous tissue disorders	146	3.90	0.71(0.61,0.84)	0.73(0.62,0.85)	−0.46(−0.70)	−0.46(−0.74)	0.73(0.61)
Endocrine disorders	118	3.15	12.80(10.66,15.38)	12.43(10.41,14.85)	3.63(3.23)	3.57(3.26)	12.42(10.34)
Respiratory, thoracic and mediastinal disorders	85	2.27	0.47(0.38,0.58)	0.48(0.39,0.60)	−1.05(−1.36)	−1.05(−1.41)	0.48(0.39)
Neoplasms benign, malignant and unspecified (incl cysts and polyps)	84	2.24	0.85(0.68,1.05)	0.85(0.69,1.05)	−0.23(−0.55)	−0.23(−0.59)	0.85(0.69)
Vascular disorders	81	2.16	1.01(0.81,1.26)	1.01(0.82,1.26)	0.02(−0.30)	0.02(−0.35)	1.01(0.81)
Surgical and medical procedures	72	1.92	1.44(1.14,1.82)	1.43(1.14,1.80)	0.52(0.17)	0.51(0.12)	1.43(1.13)
Cardiac disorders	54	1.44	0.54(0.41,0.71)	0.55(0.42,0.71)	−0.87(−1.25)	−0.87(−1.32)	0.55(0.42)
Renal and urinary disorders	48	1.28	0.67(0.50,0.89)	0.67(0.51,0.89)	−0.57(−0.98)	−0.57(−1.05)	0.67(0.51)
Eye disorders	39	1.04	0.52(0.38,0.71)	0.52(0.38,0.72)	−0.93(−1.38)	−0.93(−1.46)	0.52(0.38)
Reproductive system and breast disorders	21	0.56	0.63(0.41,0.96)	0.63(0.41,0.96)	−0.67(−1.26)	−0.66(−1.39)	0.63(0.41)
Hepatobiliary disorders	18	0.48	0.52(0.33,0.83)	0.52(0.33,0.83)	−0.93(−1.56)	−0.91(−1.71)	0.52(0.33)
Blood and lymphatic system disorders	14	0.37	0.22(0.13,0.37)	0.22(0.13,0.37)	−2.17(−2.84)	−2.13(−3.04)	0.22(0.13)
Ear and labyrinth disorders	9	0.24	0.55(0.29,1.06)	0.55(0.29,1.06)	−0.85(−1.70)	−0.82(−1.96)	0.55(0.29)
Immune system disorders	9	0.24	0.22(0.11,0.42)	0.22(0.11,0.42)	−2.19(−2.99)	−2.13(−3.27)	0.22(0.11)
Pregnancy, puerperium and perinatal conditions	7	0.19	0.43(0.21,0.91)	0.43(0.21,0.91)	−1.20(−2.12)	−1.15(−2.45)	0.43(0.21)
Social circumstances	2	0.05	0.11(0.03,0.46)	0.11(0.03,0.46)	−3.12(−4.28)	−2.84(−5.43)	0.11(0.03)
Product issues	1	0.03	0.02(0.00,0.12)	0.02(0.00,0.12)	−5.92(−6.98)	−5.34(−9.13)	0.02(0.00)

Note 1: The number of cases refers to the number of adverse events involved under the SOC.

Note 2: Proportion = number of adverse events involved in SOC/total number of adverse events.

### Distribution of the AEs at the preferred terms (PT) level

When compared to using any single method, the precision of signal detection is notably higher when the ROR, PRR, BCPNN and MGPS methods are employed in combination compared to using any single method. Consequently, this study presented the top 30 PTs ranked according to the frequency of positive signals (Table 3) and the top 30 PTs ranked by the intensity of ROR positive signals (Table 4). All of these were detected through the combined application of these four methods. In the ranking based on the positive signal frequency of the target drug, the top five PTs were Off label use (n = 187, ROR 4.06, PRR 3.91, IC 1.97, EBGM 3.91), Fatigue (n = 177, ROR 3.92, PRR 3.79, IC 1.92, EBGM 3.78), Nausea (n = 148, ROR 3.19, PRR 3.10, IC 1.63, EBGM 3.10), Adrenal insufficiency (n = 63, ROR 100.11, PRR 98.45, IC 6.61, EBGM 97.79) and Decreased appetite (n = 62, ROR 4.28, PRR 4.22, IC 2.08, EBGM 4.22). Except for Off label use, which had the highest frequency of positive signals, the remaining PTs were mentioned in the prescribing information for Osilodrostat. In the ranking of positive signal intensity, the top five PTs were Mineralocorticoid deficiency (n = 3, ROR 3351.62, PRR 3348.94, IC 11.41, EBGM 2721.20), Cortisol free urine increased (n = 6, ROR 1645.51, PRR 1642.87, IC 10.53, EBGM 1475.90), Blood corticotrophin increased (n = 11, ROR 748.14, PRR 745.95, IC 9.47, EBGM 709.53), Cortisol decreased (n = 45, ROR 466.13, PRR 460.54, IC 8.80, EBGM 446.40) and Cortisol increased (n = 19, ROR 420.53, PRR 418.41, IC 8.67, EBGM 406.71). It is noteworthy that although these PTs had a lower frequency, their corresponding positive signal intensity was significantly higher than that of other PTs, potentially representing new and potential AE signals. The adverse events associated with Oseltrolizumab and Cushing’s syndrome are depicted in S2 Fig.

**Table 3:** The top 30 PTs ranked by the frequency of positive signals.

Preferred Terms	Number of cases	ROR (95% CI)	PRR (Chi-Square)	IC (IC-2SD)	EBGM (EBGM 05)
Off label use	187	4.06 (3.50-4.70)	3.91 (409.61)	1.97 (1.73)	3.91 (3.37)
Fatigue	177	3.92 (3.37-4.56)	3.79 (367.23)	1.92 (1.68)	3.78 (3.25)
Nausea	148	3.19 (2.70-3.76)	3.10 (213.34)	1.63 (1.37)	3.10 (2.63)
Adrenal insufficiency	63	100.11 (77.98-128.53)	98.45 (6036.78)	6.61 (4.92)	97.79 (76.17)
Decreased appetite	62	4.28 (3.33-5.50)	4.22 (152.96)	2.08 (1.64)	4.22 (3.28)
Cortisol decreased	45	466.13 (345.83-628.28)	460.54 (20000.2)	8.80 (4.95)	446.40 (331.19)
COVID-19	42	3.93 (2.90-5.33)	3.90 (90.81)	1.96 (1.43)	3.90 (2.88)
Blood potassium decreased	36	19.57 (14.09-27.17)	19.39 (627.25)	4.28 (3.22)	19.36 (13.94)
Myalgia	32	3.09 (2.18-4.38)	3.07 (44.83)	1.62 (1.03)	3.07 (2.17)
Blood pressure increased	30	3.21 (2.24-4.60)	3.20 (45.39)	1.68 (1.06)	3.20 (2.23)
Oedema peripheral	24	3.12 (2.09-4.66)	3.10 (34.31)	1.63 (0.94)	3.10 (2.08)
Cortisol increased	19	420.53 (266.22-664.31)	418.41 (7690.13)	8.67 (3.60)	406.71 (257.46)
Heart rate increased	19	3.16 (2.01-4.96)	3.15 (27.89)	1.65 (0.86)	3.15 (2.01)
Oedema	16	4.90 (3.00-8.00)	4.88 (49.40)	2.29 (1.29)	4.88 (2.99)
Hypokalaemia	15	5.45 (3.28-9.04)	5.43 (54.19)	2.44 (1.36)	5.43 (3.27)
Neoplasm malignant	15	3.76 (2.26-6.24)	3.75 (30.24)	1.91 (0.95)	3.75 (2.26)
Blood sodium decreased	14	12.18 (7.21-20.59)	12.14 (143.04)	3.60 (2.05)	12.13 (7.18)
Blood potassium increased	13	13.07 (7.58-22.54)	13.03 (144.28)	3.70 (2.04)	13.02 (7.55)
Fluid retention	13	4.15 (2.41-7.15)	4.14 (30.97)	2.05 (0.98)	4.14 (2.40)
Adrenocortical insufficiency acute	12	117.80 (66.69-208.10)	117.43 (1374.15)	6.86 (2.75)	116.49 (65.95)
Blood glucose decreased	12	3.98 (2.26-7.02)	3.97 (26.70)	1.99 (0.89)	3.97 (2.25)
Blood corticotrophin increased	11	748.14 (407.80-1372.52)	745.95 (7783.38)	9.47 (2.71)	709.53 (386.75)
Neoplasm progression	10	4.44 (2.39-8.25)	4.43 (26.54)	2.15 (0.88)	4.43 (2.38)
Product administration interrupted	9	21.66 (11.26-41.68)	21.61 (176.66)	4.43 (1.90)	21.58 (11.21)
Therapeutic product effect prolonged	7	117.52 (55.82-247.42)	117.30 (800.72)	6.86 (1.89)	116.37 (55.27)
Brain fog	7	15.97 (7.60-33.53)	15.94 (97.90)	3.99 (1.45)	15.92 (7.58)
Cortisol free urine increased	6	1645.51 (706.94-3830.15)	1642.87 (8844.05)	10.53 (1.65)	1475.90 (634.08)
Blood magnesium decreased	5	9.60 (3.99-23.08)	9.59 (38.44)	3.26 (0.80)	9.58 (3.98)
Blood testosterone increased	5	38.06 (15.81-91.60)	38.01 (179.72)	5.24 (1.23)	37.91 (15.75)
Hair growth abnormal	5	11.70 (4.86-28.13)	11.68 (48.82)	3.55 (0.89)	11.68 (4.86)

**Table 4:** The top 30 PTs ranked by the intensity of positive signals.

Preferred Terms	Number of cases	ROR (95% CI)	PRR (Chi-Square)	IC (IC-2SD)	EBGM (EBGM 05)
Mineralocorticoid deficiency	3	3351.62 (954.69-11766.5)	3348.94 (8158.16)	11.41 (0.39)	2721.20 (775.12)
Cortisol free urine increased	6	1645.51 (706.94-3830.15)	1642.87 (8844.05)	10.53 (1.65)	1475.90 (634.08)
Blood corticotrophin increased	11	748.14 (407.80-1372.52)	745.95 (7783.38)	9.47 (2.71)	709.53 (386.75)
Cortisol decreased	45	466.13 (345.83-628.28)	460.54 (20000.2)	8.80 (4.95)	446.40 (331.19)
Cortisol increased	19	420.53 (266.22-664.31)	418.41 (7690.13)	8.67 (3.60)	406.71 (257.46)
Cortisol abnormal	4	283.46 (105.33-762.89)	283.16 (1103.14)	8.12 (0.99)	277.76 (103.21)
Salt craving	3	236.80 (75.64-741.34)	236.61 (692.55)	7.86 (0.52)	232.83 (74.37)
Hyperadrenocorticism	4	189.28 (70.55-507.82)	189.08 (738.73)	7.54 (0.99)	186.66 (69.58)
Glucocorticoid deficiency	4	185.06 (68.99-496.43)	184.87 (722.29)	7.51 (0.99)	182.55 (68.05)
Adrenal gland cancer	5	183.94 (76.09-444.64)	183.70 (897.16)	7.50 (1.36)	181.41 (75.05)
Adrenocortical insufficiency acute	12	117.80 (66.69-208.10)	117.43 (1374.15)	6.86 (2.75)	116.49 (65.95)
Therapeutic product effect prolonged	7	117.52 (55.82-247.42)	117.30 (800.72)	6.86 (1.89)	116.37 (55.27)
Adrenal insufficiency	63	100.11 (77.98-128.53)	98.45 (6036.78)	6.61 (4.92)	97.79 (76.17)
Steroid withdrawal syndrome	5	83.71 (34.73-201.73)	83.59 (405.70)	6.38 (1.32)	83.12 (34.49)
Hirsutism	5	60.25 (25.02-145.09)	60.17 (289.72)	5.91 (1.29)	59.92 (24.88)
Hypertrichosis	5	41.76 (17.35-100.51)	41.70 (198.06)	5.38 (1.24)	41.58 (17.28)
Blood testosterone increased	5	38.06 (15.81-91.60)	38.01 (179.72)	5.24 (1.23)	37.91 (15.75)
Blood potassium abnormal	4	25.70 (9.63-68.57)	25.67 (94.69)	4.68 (0.82)	25.63 (9.61)
Product administration interrupted	9	21.66 (11.26-41.68)	21.61 (176.66)	4.43 (1.90)	21.58 (11.21)
Blood potassium decreased	36	19.57 (14.09-27.17)	19.39 (627.25)	4.28 (3.22)	19.36 (13.94)
Cushingoid	3	16.15 (5.20-50.13)	16.14 (42.55)	4.01 (0.31)	16.12 (5.19)
Brain fog	7	15.97 (7.60-33.53)	15.94 (97.90)	3.99 (1.45)	15.92 (7.58)
Blood potassium increased	13	13.07 (7.58-22.54)	13.03 (144.28)	3.70 (2.04)	13.02 (7.55)
Cardiac flutter	5	12.75 (5.30-30.66)	12.73 (54.02)	3.67 (0.93)	12.72 (5.29)
Blood sodium decreased	14	12.18 (7.21-20.59)	12.14 (143.04)	3.60 (2.05)	12.13 (7.18)
Hair growth abnormal	5	11.70 (4.86-28.13)	11.68 (48.82)	3.55 (0.89)	11.68 (4.86)
Lung neoplasm	3	10.80 (3.48-33.52)	10.79 (26.64)	3.43 (0.20)	10.79 (3.48)
Blood magnesium decreased	5	9.60 (3.99-23.08)	9.59 (38.44)	3.26 (0.80)	9.58 (3.98)
Breast mass	3	7.28 (2.35-22.58)	7.27 (16.22)	2.86 (0.06)	7.27 (2.34)
Parosmia	3	6.58 (2.12-20.42)	6.58 (14.18)	2.72 (0.01)	6.57 (2.12)

ROR, reported odds ratio; IC, information component; EBGM, the empirical Bayes geometric mean; IC025 and EBGM05, lower limit of the 95% two-sided confidence interval for IC and EBGM, respectively. Signals are detected when all the following criteria are met: PRR ≥ 2 and χ2 > 4, lower limit of 95% CI of ROR > 1, IC025 > 0, EBGM05 > 2.

### Time to event of the AEs

**Fig 3:**
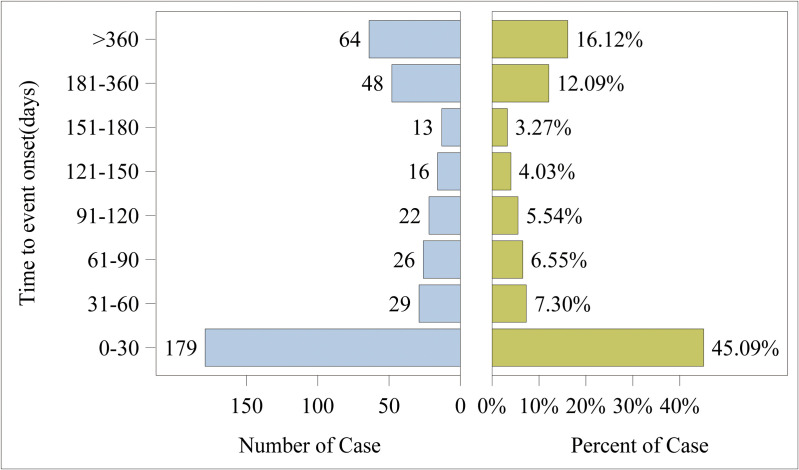
The distribution of the onset time for the adverse events associated with Osilodrostat.

**Fig 4:**
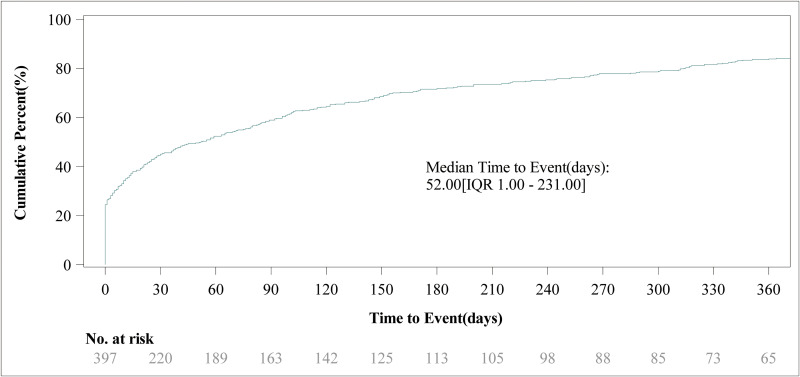
The cumulative incidence of adverse events related to Osilodrostat over time.

**Fig 5:**
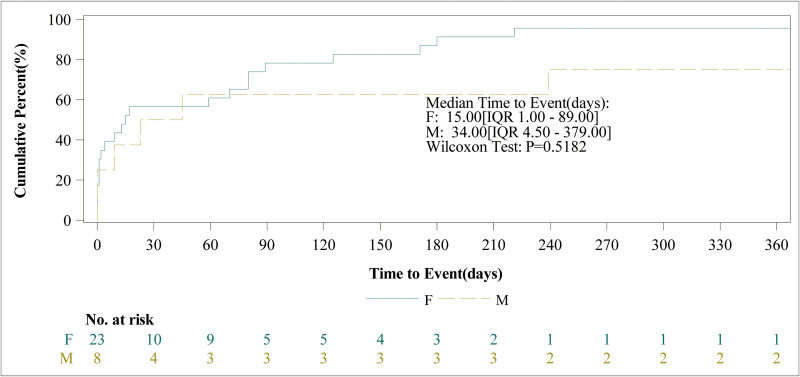
Osilodrostat adverse event incidence over time by sex.

After excluding reports with incomplete records of the AEs’ occurrence time, we analyzed all the 397 reports that had detailed records of AEs occurrence time. As shown in Fig 3, nearly half of the AEs (45.09%) occurred within 0–30 days after the use of Osilodrostat. A significant number of AEs occurred between 181–360 days and after 360 days of Osilodrostat use. The proportion of AEs in these three time periods was considerably larger than that in other time periods. Fig 4 illustrated the cumulative incidence of to Osilodrostat – related over time. Moreover, there was a difference in the median occurrence time of AEs between males and females (Fig 5). Analysis using the Weibull distribution indicated an early-failure mode. The detailed parameters of this analysis are presented in Table 5.

**Table 5:** Time to onset of Osilodrostat-associated adverse events and Weibull distribution analysis.

		Weibull distribution				
Cases	TTO (days)	Scale parameter		Shape parameter		
n	median(IQR)	α	95% CI	β	95% CI	Failure type
397	52.00(1.00,231.00)	187.21	158.80 - 220.69	0.73	0.66 - 0.79	Early failure

## Discussion

In this study, data from the FAERS database was utilized to evaluate the safety of Osilodrostat, with the objective of furnishing recommendations for its clinical utilization.

The results indicated that the AEs related to Osilodrostat were more prevalent in women. This could potentially be attributed to the inhibitory effect of Osilodrostat on CYP11B1. Such inhibition leads to the accumulation of 11 – deoxycorticosterone and classic androgen precursors like DHEA and A4, which in turn promote androgen synthesis [6]. Excessive androgens can cause acne and hirsutism in women [24]. Furthermore, the observed gender difference might also be related to the fact that the majority of reports submitted to the FDA were from the consumers (78.29%). Socially, women tend to be more proactive in voicing their concerns and are thus more likely to report AEs [25]. Although some women may experience acne and hirsutism upon initiating Osilodrostat treatment, studies have shown that with extended follow-up, the levels of androgens in female patients gradually return to baseline levels, leading to progressive improvement in symptoms [5,6,26,27]. Therefore, in clinical practice, for patients experiencing acne and hirsutism, local treatments or the use of spironolactone to block the androgen receptor activity can be considered [28]. It is also crucial to reassure patients that these symptoms are transient to ease their concerns. The majority of reports come from the North America and Europe, likely because the EMA and FDA were the first regulatory agencies to approve Osilodrostat for the treatment of CS [2,3]. The number of the AEs reported in 2024 far exceeded those in other years, suggesting that Osilodrostat was being increasingly used in clinical settings. However, the proportion of severe AEs and hospitalizations caused by Osilodrostat remained high, indicating that vigilance should not be relaxed and monitoring of Osilodrostat-related AEs should be strengthened [29].

Through disproportionality analysis, we found that Investigations and Gastrointestinal disorders were not only among the top five most common AEs related to Osilodrostat across the entire SOC hierarchy, but also among the top five with the strongest signals. Gastrointestinal disorders have been mentioned in the drug’s package insert and multiple clinical studies [5,7,9], which validates the reliability of our findings. The use of Osilodrostat can lead to gastrointestinal symptoms such as nausea, vomiting, and diarrhea. This is associated with the rapid blockade of adrenal enzymes by Osilodrostat, resulting in a sudden decrease in cortisol and subsequent hypocortisolism [30,31]. However, Investigations were rarely mentioned. This might because Investigations represented the outcomes of the AEs. Patients using Osilodrostat undergo various examinations when AEs occur, which increased the frequency and signal strength of this SOC. However, in these cases, Investigations were not typically mentioned separately in drug package inserts or clinical studies.

In terms of PT analysis, our study found that Off label use, Fatigue, Nausea, and Adrenal insufficiency were the most frequently reported terms, and they also had the highest frequency of positive signals. The occurrence of Fatigue, Nausea, and Adrenal insufficiency was not surprising, as these PTs had been mentioned in numerous clinical studies and were also included in the drug information for Osilodrostat [5,7,9]. Additionally, Fatigue and Nausea corresponded to the most frequently occurring SOC, Gastrointestinal disorders.

The five PTs with the strongest signals were Mineralocorticoid deficiency, Cortisol free urine increased, Blood corticotrophin increased, Cortisol decreased, and Cortisol increased. The occurrence of these PTs was closely related to the pharmacological effects of Osilodrostat, as it regulated both glucocorticoid and mineralocorticoid levels in the body [32,33]. It is a dual inhibitor of the biosynthetic pathways for glucocorticoids and mineralocorticoids [33]. Inappropriate dosing can lead to abnormal levels of these hormones, which can have serious consequences [34]. Mineralocorticoid deficiency can cause hyponatremia, hyperkalemia, low blood pressure, and adrenal crisis [35,36]. Increased cortisol levels can lead to uncontrolled or worsening symptoms of CS, while decreased cortisol levels can result in hypotension, hypoglycemia, fatigue, dizziness, decreased appetite, nausea, vomiting, diarrhea, abdominal pain, and even syncope and adrenal crisis [37,38]. This provides two important insights. First, when using Osilodrostat clinically, it is crucial to strictly follow the standardized application procedures for the drug. Second, monitoring of mineralocorticoid and cortisol levels should be conducted during its use. It is particularly important to note that when measuring cortisol levels in patients, laboratory methods with minimal cross-reactivity with cortisol precursors (such as 11-deoxycorticosterone) should be prioritized to avoid overestimating cortisol levels [26,39]. Additionally, cortisol levels can fluctuate during fever, infection, or increased psychological stress, so more frequent and careful monitoring of cortisol levels should be conducted in these situations [40].

This study also analyzed the timing of the AEs. The median time for the occurrence of the AEs was 52 days after the medication use. However, subgroup analysis revealed differences in the median time of the AEs between males and females, with males experiencing them at a median of 15 days and females at 34 days. The reasons for this difference have not yet been elucidated by research. We should conduct more frequent and close monitoring of AEs in patients around these time points. Additionally, patients should be informed to pay attention to any discomfort they experienced around these time points and reported it promptly to their healthcare professionals. Through these measures, the AEs can be detected in a timely manner and interventions can be initiated early to ensure the patients’ safety.

In contrast to the previously available specification for Osilodrostat, we found that the appearance of off label use was unexpected, suggesting that future efforts should be focused on strengthening the supervision and management of Osilodrostat use and standardizing the procedures for healthcare professionals [41]. Furthermore, patient health education should emphasize the importance of following medical advice when taking medication. In addition, we found that “decreased appetite” appeared much more frequently in this study than in the drug package inserts. Similar to our study, Gadelha M et al. [9] also highlighted decreased appetite as a high incidence of adverse events in their study. This suggests that we still need to pay extra attention to the occurrence of decreased appetite in patients using Osilodrostat in the future and be prepared to provide additional nutritional support to patients using Osilodrostat to avoid nutritional imbalance caused by decreased appetite.

The strength of this study lies in its ability to conduct a large-sample analysis using the FAERS database, allowing for a comprehensive evaluation of the safety of Osilodrostat. However, the study inevitably has some limitations. Some of these limitations stem from the FAERS database itself [42], such as incomplete reports, underreporting, and selective reporting, which introduce biases. Additionally, the FAERS database does not provide certain clinical information about patients, such as comorbidities and medications being used, making it difficult to control for confounding factors [43]. Lastly, while disproportionality analysis assesses signal strength and establishes statistical associations, it does not provide sufficient evidence to determine the causal relationship between drug use and the AEs [44]. In future studies, high-quality randomized controlled trials (RCTs) could be added to confirm causal relationships.

## Conclusion

We conducted a large-sample study using the FAERS database to comprehensively assess the safety of Osilodrostat. At the SOC level, Investigations and Gastrointestinal disorders were the most common and strongest signals of the AEs associated with Osilodrostat. At the PT level, Off-label use, Fatigue, Nausea, and Adrenal insufficiency were the most frequently reported AEs with the highest number of positive signals related to Osilodrostat. However, due to the inherent limitations of the FAERS database and the analysis methods used, biases may be introduced, confounding factors cannot be ruled out, and causality cannot be confirmed. Therefore, the results of this study should be interpreted and applied with caution. Additionally, future high-quality randomized controlled trials (RCTs) could be conducted to confirm the association between Osilodrostat and these AEs.

## Supporting information

S1 Fig The percentage of Osilodrostat use in five countries and the incidence of hospitalization, death, life-threatening events, and disability. A:the percentage of Osilodrostat use in five countries; B: the incidence of hospitalization, death, life-threatening events, and disability.
(TIF)

S2 Fig The the adverse events related to Cushing’s syndrome associated with Osilodrostat use. (TIF)

## References

[1] Fleseriu M, Pivonello R, Young J, Hamrahian AH, Molitch ME, Shimizu C, et al. Osilodrostat, a potent oral 11β-hydroxylase inhibitor: 22-week, prospective, phase II study in Cushing’s disease. Pituitary. 2016;19(2):138–48. doi: 10.1007/s11102-015-0692-z 26542280 PMC4799251

[2] Duggan S. Osilodrostat: first approval. Drugs. 2020;80(5):495–500. 32141023 10.1007/s40265-020-01277-0

[3] Rasool S, Skinner BW. Osilodrostat for the treatment of Cushing’s disease. Expert Opin Pharmacother. 2021;22(9):1099–106. doi: 10.1080/14656566.2021.1897106 33703978

[4] Fleseriu M. Medical treatment of Cushing disease: new targets, new hope. Endocrinol Metab Clin North Am. 2015;44(1):51–70. doi: 10.1016/j.ecl.2014.10.006 25732642

[5] Pivonello R, Fleseriu M, Newell-Price J, Bertagna X, Findling J, Shimatsu A, et al. Efficacy and safety of osilodrostat in patients with Cushing’s disease (LINC 3): a multicentre phase III study with a double-blind, randomised withdrawal phase. Lancet Diabetes Endocrinol. 2020;8(9):748–61. doi: 10.1016/S2213-8587(20)30240-0 32730798

[6] Fleseriu M, Newell-Price J, Pivonello R, Shimatsu A, Auchus RJ, Scaroni C, et al. Long-term outcomes of osilodrostat in Cushing’s disease: LINC 3 study extension. Eur J Endocrinol. 2022;187(4):531–41. doi: 10.1530/EJE-22-0317 35980235 PMC9513654

[7] Bertagna X, Pivonello R, Fleseriu M, Zhang Y, Robinson P, Taylor A, et al. LCI699, a potent 11β-hydroxylase inhibitor, normalizes urinary cortisol in patients with Cushing’s disease: results from a multicenter, proof-of-concept study. J Clin Endocrinol Metab. 2014;99(4):1375–83. doi: 10.1210/jc.2013-2117 24423285

[8] Fleseriu M, Biller BMK, Bertherat J, Young J, Hatipoglu B, Arnaldi G, et al. Long-term efficacy and safety of osilodrostat in Cushing’s disease: final results from a Phase II study with an optional extension phase (LINC 2). Pituitary. 2022;25(6):959–70. doi: 10.1007/s11102-022-01280-6 36219274 PMC9675663

[9] Gadelha M, Bex M, Feelders RA, Heaney AP, Auchus RJ, Gilis-Januszewska A, et al. Randomized Trial of Osilodrostat for the treatment of cushing disease. J Clin Endocrinol Metab. 2022;107(7):e2882–95. doi: 10.1210/clinem/dgac178 35325149 PMC9202723

[10] Tanaka T, Satoh F, Ujihara M, Midorikawa S, Kaneko T, Takeda T, et al. A multicenter, phase 2 study to evaluate the efficacy and safety of osilodrostat, a new 11β-hydroxylase inhibitor, in Japanese patients with endogenous Cushing’s syndrome other than Cushing’s disease. Endocr J. 2020;67(8):841–52. doi: 10.1507/endocrj.EJ19-0617 32378529

[11] Detomas M, Altieri B, Deutschbein T, Fassnacht M, Dischinger U. Metyrapone versus osilodrostat in the short-term therapy of endogenous cushing’s syndrome: results from a single center cohort study. Front Endocrinol. 2022;13:903545. 10.3389/fendo.2022.903545 PMC9235400 35769081

[12] Ou Y, Cui Z, Lou S, Zhu C, Chen J, Zhou L, et al. Analysis of tirzepatide in the US FDA adverse event reporting system (FAERS): a focus on overall patient population and sex-specific subgroups. Front Pharmacol. 2024;15:1463657. doi: 10.3389/fphar.2024.1463657 39568578 PMC11576270

[13] Wang Y, Zhao B, Yang H, Wan Z. A real-world pharmacovigilance study of FDA adverse event reporting system events for sildenafil. Andrology. 2024;12(4):785–92. doi: 10.1111/andr.13533 37724699

[14] Zhao B, Fu Y, Cui S, Chen X, Liu S, Luo L. A real-world disproportionality analysis of Everolimus: data mining of the public version of FDA adverse event reporting system. Front Pharmacol. 2024;15:1333662. doi: 10.3389/fphar.2024.1333662 38533254 PMC10964017

[15] Yang H, Wan Z, Chen M, Zhang X, Cui W, Zhao B. A real-world data analysis of topotecan in the FDA Adverse Event Reporting System (FAERS) database. Expert Opin Drug Metab Toxicol. 2023;19(4):217–23. doi: 10.1080/17425255.2023.2219390 37243615

[16] Zhao B, Zhang X, Chen M, Wang Y. A real-world data analysis of acetylsalicylic acid in FDA Adverse Event Reporting System (FAERS) database. Expert Opin Drug Metab Toxicol. 2023;19(6):381–7. doi: 10.1080/17425255.2023.2235267 37421631

[17] Zhong C, Zheng Q, Zhao B, Ren T. A real-world pharmacovigilance study using disproportionality analysis of United States food and drug administration adverse event reporting system events for vinca alkaloids: comparing vinorelbine and vincristine. Expert Opin Drug Saf. 2024;23(11):1427–37. 39340205 10.1080/14740338.2024.2410436

[18] Jiang Y, Lu R, Du Z, Shen Y, Zhou Q, Luan P, et al. The real-world safety assessment of Siponimod: a systematic analysis based on the FAERS database. J Neurol Sci. 2025;468:123364. doi: 10.1016/j.jns.2024.123364 39732042

[19] Zhang Y, Deng W, Wang M, Luo S, Li S. A real-world pharmacovigilance study of neuroleptic malignant syndrome based on FDA adverse event reporting system. Front Pharmacol. 2024;15:1438661. doi: 10.3389/fphar.2024.1438661 39723245 PMC11668602

[20] Rothman KJ, Lanes S, Sacks ST. The reporting odds ratio and its advantages over the proportional reporting ratio. Pharmacoepidemiol Drug Saf. 2004;13(8):519–23. doi: 10.1002/pds.1001 15317031

[21] Evans SJ, Waller PC, Davis S. Use of proportional reporting ratios (PRRs) for signal generation from spontaneous adverse drug reaction reports. Pharmacoepidemiol Drug Saf. 2001;10(6):483–6. doi: 10.1002/pds.677 11828828

[22] Rivkees SA, Szarfman A. Dissimilar hepatotoxicity profiles of propylthiouracil and methimazole in children. J Clin Endocrinol Metab. 2010;95(7):3260–7. doi: 10.1210/jc.2009-2546 20427502

[23] Ang PS, Chen Z, Chan CL, Tai BC. Data mining spontaneous adverse drug event reports for safety signals in Singapore - a comparison of three different disproportionality measures. Expert Opin Drug Saf. 2016;15(5):583–90. doi: 10.1517/14740338.2016.1167184 26996192

[24] Dormoy A, Haissaguerre M, Vitellius G, Do Cao C, Geslot A, Drui D, et al. Efficacy and safety of osilodrostat in paraneoplastic cushing syndrome: a real-world multicenter study in France. J Clin Endocrinol Metab. 2023;108(6):1475–87. doi: 10.1210/clinem/dgac691 36470583 PMC10188310

[25] Lee KMN, Rushovich T, Gompers A, Boulicault M, Worthington S, Lockhart JW, et al. A gender hypothesis of sex disparities in adverse drug events. Soc Sci Med. 2023;339:116385. doi: 10.1016/j.socscimed.2023.116385 37952268

[26] Pivonello R, Simeoli C, Di Paola N, Larocca A, Crescenzo EM, Colao A. Osilodrostat: a novel potent inhibitor of 11-beta-hydroxylase for the treatment of Cushing’s syndrome. touchREV Endocrinol. 2024;20(1):43–51. doi: 10.17925/EE.2024.20.1.8 38812665 PMC11132648

[27] Perosevic M, Tritos NA. Clinical utility of osilodrostat in Cushing’s disease: review of currently available literature. Drug Des Devel Ther. 2023;17:1303–12. doi: 10.2147/DDDT.S315359 37143705 PMC10151255

[28] Fleseriu M, Biller BMK. Treatment of Cushing’s syndrome with osilodrostat: practical applications of recent studies with case examples. Pituitary. 2022;25(6):795–809. doi: 10.1007/s11102-022-01268-2 36002784 PMC9401199

[29] Zhang Z, Yao Y, Zhu L. Assessing real-world safety of plecanatide: a pharmacovigilance study based on the FDA adverse event reporting system. Front Pharmacol. 2024;15:1500810. doi: 10.3389/fphar.2024.1500810 39654622 PMC11625541

[30] Shimatsu A, Biller BM, Fleseriu M, Pivonello R, Lee EJ, Leelawattana R, et al. Osilodrostat treatment in patients with Cushing’s disease of Asian or non-Asian origin: a pooled analysis of two Phase III randomized trials (LINC 3 and LINC 4). Endocr J. 2024;71(12):1103–23. doi: 10.1507/endocrj.EJ24-0153 39183039 PMC11778389

[31] Simões Corrêa Galendi J, Correa Neto ANS, Demetres M, Boguszewski CL, Nogueira VDSN. Effectiveness of medical treatment of Cushing’s disease: a systematic review and meta-analysis. Front Endocrinol (Lausanne). 2021;12:732240. doi: 10.3389/fendo.2021.732240 34603209 PMC8485729

[32] Gadelha M, Snyder PJ, Witek P, Bex M, Belaya Z, Turcu AF, et al. Long-term efficacy and safety of osilodrostat in patients with Cushing’s disease: results from the LINC 4 study extension. Front Endocrinol (Lausanne). 2023;14:1236465. doi: 10.3389/fendo.2023.1236465 37680892 PMC10482037

[33] Castinetti F. Pharmacological treatment of Cushing’s syndrome. Arch Med Res. 2023;54(8):102908. doi: 10.1016/j.arcmed.2023.102908 37977919

[34] Yuen KCJ. Osilodrostat: a review of recent clinical studies and practical recommendations for its use in the treatment of cushing disease. Endocr Pract. 2021;27(9):956–65. doi: 10.1016/j.eprac.2021.06.012 34389514

[35] Heidarpour M, Shafie D, Eshraghi R, Mirjalili SR, Bahrami A, Movahed MR. Adrenal crisis-induced cardiogenic shock (ACCS): a comprehensive review. Heart Fail Rev. 2025;30(1):227–46. doi: 10.1007/s10741-024-10458-y 39503801

[36] Sun K, Wang Y-L, Hou C-C, Shang D, Du L-J, Bai L, et al. Collecting duct NCOR1 controls blood pressure by regulating mineralocorticoid receptor. J Adv Res. 2025;68:75–87. doi: 10.1016/j.jare.2024.02.003 38341030 PMC11785564

[37] Regazzo D, Mondin A, Scaroni C, Occhi G, Barbot M. The role of glucocorticoid receptor in the pathophysiology of pituitary corticotroph adenomas. Int J Mol Sci. 2022;23(12):6469. doi: 10.3390/ijms23126469 35742910 PMC9224504

[38] Courties G, Frodermann V, Honold L, Zheng Y, Herisson F, Schloss MJ, et al. Glucocorticoids regulate bone marrow B lymphopoiesis after stroke. Circ Res. 2019;124(9):1372–85. doi: 10.1161/CIRCRESAHA.118.314518 30782088 PMC6483874

[39] Nagendra L, Dutta D, Raizada N, Surana V, Selvan C, Bhattacharya S. Efficacy and safety of osilodrostat in managing cushing’s syndrome: a systematic review and meta-analysis. Indian J Endocrinol Metab. 2024;28(3):232–8. doi: 10.4103/ijem.ijem_260_23 39086571 PMC11288521

[40] Seizer L. Anticipated stress predicts the cortisol awakening response: an intensive longitudinal pilot study. Biol Psychol. 2024;192:108852. 39102975 10.1016/j.biopsycho.2024.108852

[41] Kotalczyk A, Guo Y, Wang Y, Lip GY. Are low doses of non-vitamin K antagonists effective in Chinese patients with atrial fibrillation? A report from the optimal thromboprophylaxis in elderly Chinese patients with atrial fibrillation (Chioteaf) registry. Int J Stroke. 2021. doi: 10.1177/17474930211068785236 34657532

[42] Li B, Hu X, Yue Z. Drug-induced hearing disorders: a disproportionality analysis of the FAERS database. Front Pharmacol. 2024;15:1480994. doi: 10.3389/fphar.2024.1480994 39650160 PMC11620887

[43] Liu Q, Cui Z, Deng C, Yang C, Shi T. A real-world pharmacovigilance analysis of adverse events associated with irbesartan using the FAERS and JADER databases. Front Pharmacol. 2024;15:1485190. doi: 10.3389/fphar.2024.1485190 39635439 PMC11614654

[44] Barcelos FC, de Matos GC, da Silva MJS, da Silva FAB, Lima E da C. Suspected adverse drug reactions related to breast cancer chemotherapy: disproportionality analysis of the brazilian spontaneous reporting system. Front Pharmacol. 2019;10:498. doi: 10.3389/fphar.2019.00498 31139083 PMC6519311

