# Supplementary figures and images for: Safety assessment of Osilodrostat: The adverse event analysis based on FAERS database by means of disproportionality analysis
Source: PLoS One. 2025 Aug 7;20(8):e0329088. doi: 10.1371/journal.pone.0329088 (PMC12331061; doi:10.1371/journal.pone.0329088)

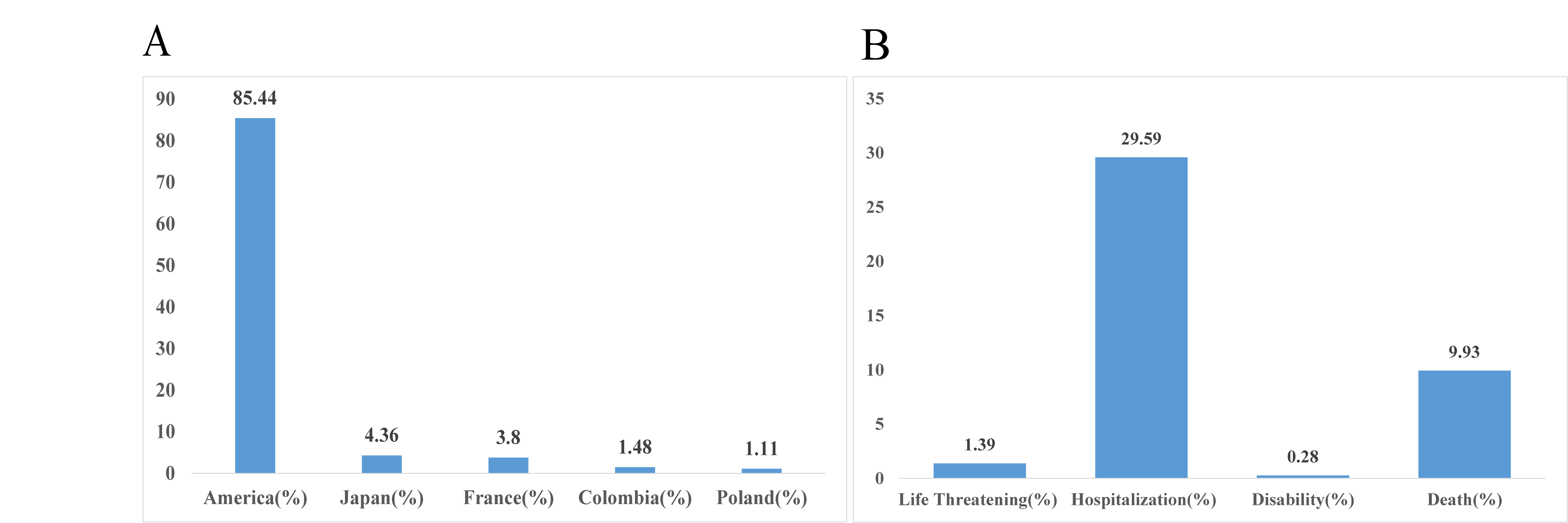

Supplement: S1 Fig — A:the percentage of Osilodrostat use in five countries; B: the incidence of hospitalization, death, life-threatening events, and disability. (TIF) [file pone.0329088.s001.tif]

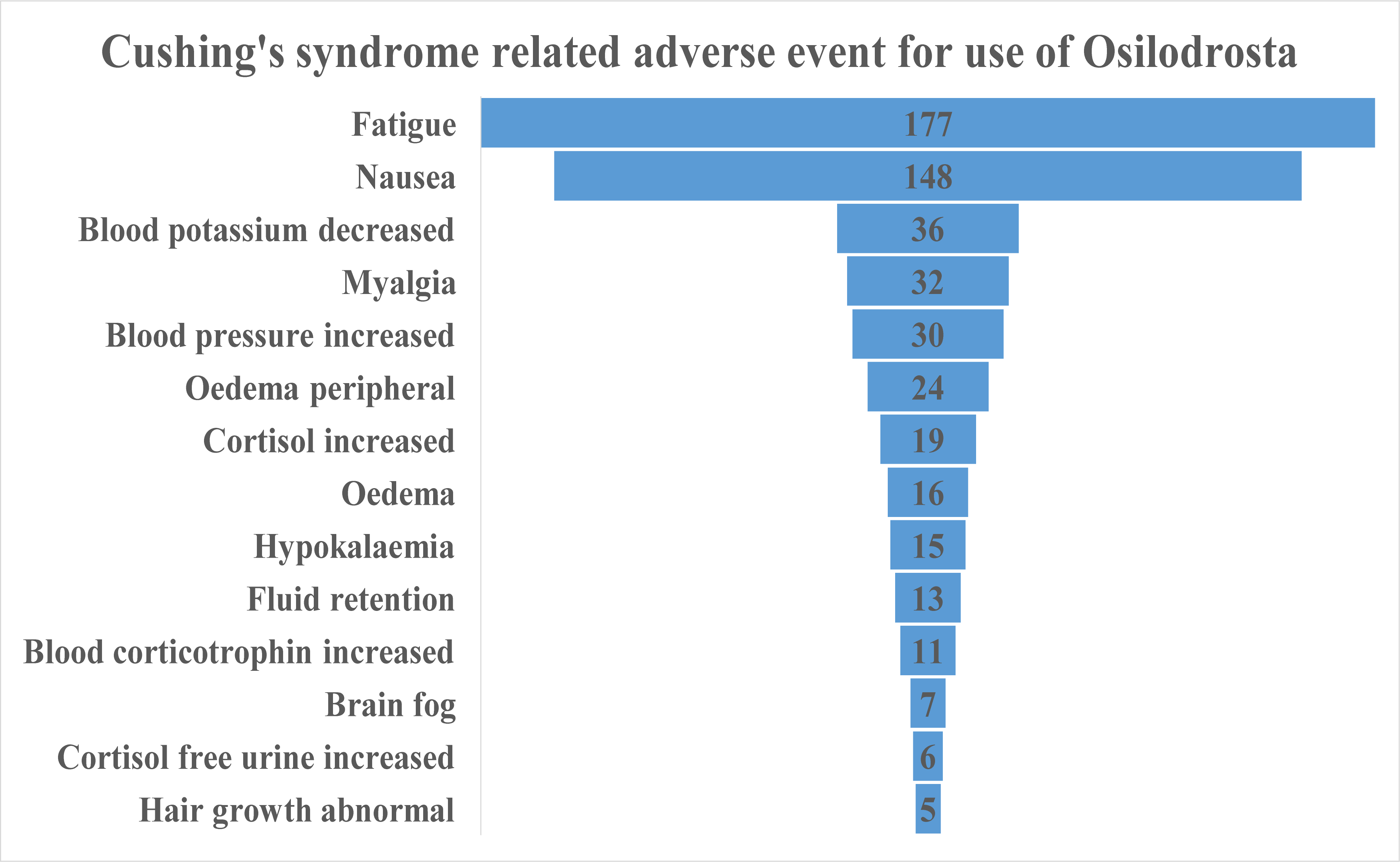

Supplement: S2 Fig — (TIF) [file pone.0329088.s002.tif]
